# Income inequality and self-rated health status in Colombia

**DOI:** 10.1186/s12939-022-01659-8

**Published:** 2022-05-16

**Authors:** Pamela Góngora-Salazar, María Sofía Casabianca, Paul Rodríguez-Lesmes

**Affiliations:** 1 Nuffield Department of Population Health, Health Economics Research Centre, University of Oxford, Richard Doll Building, Old Road Campus, Oxford, OX3 7LF UK; 2 Alianza EFI, Universidad del Rosario, Calle 12 C No 4 -69, Bogotá, 111711 Colombia; 3 School of Economics, Universidad del Rosario, Calle 12 C No. 4 – 69, 111711 Bogotá, Colombia

**Keywords:** Income inequality, Self-rated health status, Health inequalities, Colombia, Income

## Abstract

**Background:**

The negative association between income inequality and health has been known in the literature as the Income Inequality Hypothesis (IIH). Despite the multiple studies examining the validity of this hypothesis, evidence is still inconclusive, and the debate remains unsolved. In addition, relatively few studies have focused their attention on developing or emerging economies, where levels of inequality tend to be the highest in the world. This work examines the statistical association between income inequality and self-rated health status in Colombia, a highly unequal Latin American country.

**Methods:**

To explore whether this association is present in the general population or whether it is only confined to the bottom of the income distribution, we use data from the 2011–2019 National Quality of Life Survey. Multiple probit estimations are considered for testing the robustness of the IIH.

**Results:**

Evidence favouring the IIH was found, even after controlling for individual income levels, average regional income, and socioeconomic characteristics. The link between income inequality and the probability of reporting poor health seems to be present across all income quintiles. However, the magnitude of such association is considerably smaller when using inequality measures with relatively greater sensitivity to income differences among the rich.

**Conclusions:**

The association between regional income inequality and individual's self-rated health status in Colombia is not only confined to low-income individuals but extends across all socioeconomic strata. This association is robust to the income inequality measure implemented, the income-unit of analysis, and changes in the sample. It is suggested that reducing income disparities can potentially contribute to improving individual's health.

**Supplementary Information:**

The online version contains supplementary material available at 10.1186/s12939-022-01659-8.

## Background

The historically high levels of income inequality have been a matter of concern due to its moral dimensions and the impact on society's well-being. Income inequality has been shown to have adverse effects on social capital [1, 2], education attainment, and economic growth [3]. Some studies have also suggested a negative causal association between income inequality and health [4]. Such association -known as the Income Inequality Hypothesis (IIH)- states that an individual's health is affected not only by the individual's own level of income but also by the level of inequality in the area of residence [5, 6]. Although various scholars have reported evidence in favour of this hypothesis, others have documented a nil relationship between income distribution and health [7].

From a policy perspective, understanding the effects of inequality on health remains as a matter of concern: "*if inequality is shown to have a lasting impact on outcomes like health, then it may be beneficial and efficient to minimize inequality instead of designing policies to correct differences in outcomes. In contrast, if inequality has little or no impact on measurable outcomes, then it will be placed in the realm of a social or moral issue rather than an economic one*" [8]. Some scholars have suggested the existence of a threshold above which income inequality affects an individual's health, meaning that it is more likely to find a negative statistical association between income disparities and health outcomes in countries with highly unequal income distributions [9]. Nevertheless, most of the studies have focused their attention on industrialized countries, and relatively few research pieces have concentrated on developing or emerging economies, which tend to report very high levels of income inequality [10].

This study aims to examine whether there is a statistical association between income inequality and self-rated health status across Colombian regions for the 2011–2019 period. With an average life expectancy of 77.3 years, and a US $5,333 GDP per-capita (current US$), the Colombian society is in an advanced stage of the epidemiological and economic transition [11]. The country is one of the most unequal nations worldwide, reporting a Gini coefficient of 0.51 for 2019, by far more unequal than the United States (0.41) [11]. Up until today, no study has evaluated the association between rated-health status and income disparities in Colombia.

We use data from the 2011–2019 National Quality of Life Survey to test this association. Several probit estimations are considered for testing the robustness of the IIH. In general, after controlling for individual's income, regional average income, and other potential cofounders, a statistical association between income inequality and health status is observed. This association is not only confined to the lowest income quintile but extends across all socioeconomic strata. The marginal effect of income inequality was found to be weaker when income inequality was operationalised with indices with relatively greater sensitivity to income differences at the top end of the distribution.

The remaining of this paper is organized as follows: Sect. 2 summarizes the findings of previous studies. Section 3 describes the data used for assessing the IIH. Section 4 explains the empirical strategy and the main results, while Sect. 5 reports some robustness checks. Sections 6 and 7 discuss the results obtained, the limitations of this work, as well as the conclusions reached.

### Relevant studies

Since the 90 s, multiple studies have been published aiming at exploring the relationship between income inequality and health [6, 7]. The first group of studies, which adopted a multiple aggregate-level approach, reported a strong association between income differences and aggregate health outcomes, favouring the IIH [12–15]. These ecological studies were however highly criticized due to their incapacity to disentangle the effects of individual income from the contextual effects of income inequality. Gravelle 1998 showed that the associations between income inequality and population health is a “*statistical artefact*” that results from the non-linear relationship between individual health and income levels [16]. In response to this critique, since 1997 multiple scholars have published multilevel-studies, based on individual-level data [4]. Findings have been however highly heterogeneous within and across countries.

Most of the IIH literature has been concentrated in the United States (US), but studies seem to be conclusive. Daly, et. al. 1998 used a Panel Study of Income Dynamics to test the association between individual mortality risk and various measures of income inequality [8]. Authors did not find any significant association, except in the case of those with middle-incomes between the ages of 25 and 64. Mellor and Milyo 2002 also failed to find evidence in favour of the IIH when using the data from the Current Population Survey (CPS). The authors explored the effect of income inequality on individual's health status for both the general population and low-income individuals [17]. This allowed them to test both the weak and strong versions of the IIH. The former postulates that income inequality negatively affects the health of all members of society, while the strong version posits that income inequality only affects the poor's health. The study controlled for both individual characteristics and regional variations in access to health services or social norms toward health. In contrast to these studies, Fiscella and Franks found that, after adjustment for age and sex, income inequality has an independent effect on the level of depressive symptoms, and on baseline and follow-up self-rated health [18].

Different explanations have been suggested to explain the discrepant results within the US. Some scholars have suggested that the apparent association between income dispersion and health might be driven by misspecification of individual income and residual confounding [19]. Some studies, however, have offered evidence in the opposite direction [4]. Other authors have also suggested the possibility of confounding by differences in educational attainment since some studies have failed to find a statically significant association when controlling for education. Evidence in this regard is, however, mixed [4]. Likewise, the possibility that racial composition confounds the income inequality-health relation has been suggested, but the literature is not conclusive [20]. The geographic scale at which income inequality is measured has also been suggested as a potential explanation. While U.S. multilevel-studies that measure income inequality at state-level tend to report a solid association between inequality and health, those that measure income inequality at lower levels of aggregation are rather non-supportive of the IIH [4, 7, 10]. There are, however, some studies that fail to find a statistical association between state-level and metropolitan-area-level inequality and individual's health status [17]. Despite the very large literature examining the validity of the IIH, evidence is still inconclusive, and the debate remains unsolved.

Most of the multilevel studies conducted outside the U.S. have failed to find evidence in favour of the IIH [4]. For Britain, authors found limited evidence of the association between regional income inequality and worse self-rated health, especially among those with the lowest incomes [21]. Across European countries, using the longitudinal data from the European Community Household Panel Survey (1994–2001), authors found statistically significant evidence supporting the IIH, though the magnitude of such effect was very small [22]. Similar findings were obtained for Canada [23], and for Japan [24].

Interestingly, most non-US studies that report a nil association between income inequality and health correspond to industrialized economies with more egalitarian income distribution than the U.S., and stronger welfare regimes. The economic and social security policies in these countries, including the provision of comprehensive and universal healthcare, make income inequality smaller and are likely to reduce the potential impact of income inequality on individuals’ health. Conversely, research pieces of countries with relatively high unequal income distributions tend to report a statistically significant relationship between income distribution and health [7]. In Chile, for instance, Subramanian and collages examined the cross-sectional multilevel associations between income inequality and self-rated poor health, finding evidence in favour of the IIH [25]. Similarly, for Brazil, another more unequal country than the U.S, authors identified a strong statistical negative association between income inequality and life expectancy, by using a panel dataset for the 27 Brazilian states over the period 2000–2009 [26]. Studies for Ecuador and for India also found a strong association between economic inequality and health [27, 28]. This has led some scholars to suspect the existence of a threshold above which income inequality affects health outcomes [4, 9].

Despite the few studies mentioned above, the IIH literature focused on low-middle-income countries is still minimal [29, 30]. More empirical studies in these countries are needed to understand under which conditions income inequality has a detrimental effect on population health. Further research could also provide a better understanding of the methodological aspects that drive the different results concerning the role of income distribution on individuals’ health.

## Methods

Data from the 2011–2019 Quality of Life National Survey (*Encuesta Nacional de Calidad de Vida* –ECV) was used to examine the income inequality-health association. Data from 2017 was not included since relevant questions for the research were not added to that year's survey. The ECV is a national, population-based survey that is carried out every year by the National Administrative Department of Statistics (DANE). The sampling information from these surveys is representative of the national total and nine big regions. Within regions (except for San Andrés, Orinoquía-Amazonas and Bogotá), the survey is representative of urban centres, hamlets and dispersed rural areas. For comparability purposes with past studies [17, 22], the sample is limited to individuals between the ages of 24 and 75. The resulting sample consists of 567,678 individual observations over eight years of the survey.

### Health indicator

Self-rated health, an overall assessment by the individual of their health status, is the most common outcome variable in the literature that studies the relationship between inequality and health [7]. It is a common measure of an individual's health. Multiple studies have found it to be a robust predictor of subsequent morbidity and mortality [31–33]. In the ECV, the self-reported health variable is measured on a 4-point scale labelled very good, good, fair and bad/poor, and individuals respond to the question: "*In general terms, the health status of respondent's name is…*?". All household members are required to answer this question, which is formulated directly to individuals aged 18 or older. Following a study in the United States, among other previous studies, this health-indicator is dichotomized with 0 for "*very good or good*" and 1 for "*fair or poor*" [17]. Dichotomizing this 4-point scale variable is a useful strategy for increasing the reliability of self-rated health in the general population [34].

In the region-level sample, on average, 80.8% of men and 73.1% of women reported being in very good or good health. This observation is consistent with the "gender paradox in health"; women tend to report higher morbidity rates than men, even though they experience greater longevity than their counterparts [35]. Table 1 shows the distribution of self-reported health by region and gender. Fair or poor health prevalence ranges from 10.9%-8.0% in San Andres to 33.1%-42.3% in Pacífico, which is the most disadvantaged region in Colombia.

**Table 1:** Distribution of Self-Reported Health Level by Region (percentages)

	**Region**	**On a 4-Point Scale**				**Fair or Poor**
		**Very Good**	**Good**	**Fair**	**Poor**	
**Men**	**Atlántica**	8.0%	75.4%	15.4%	1.2%	16.6%
	**Oriental**	7.9%	69.8%	20.6%	1.7%	22.3%
	**Central**	14.0%	63.9%	20.1%	2.0%	22.2%
	**Pacífico**	5.4%	61.5%	29.5%	3.7%	33.1%
	**Bogotá**	14.6%	70.9%	13.4%	1.1%	14.5%
	**Antioquia**	20.1%	62.9%	15.4%	1.6%	17.0%
	**Valle del Cauca**	14.5%	68.6%	15.7%	1.2%	16.9%
	**San Andrés**	11.2%	80.8%	7.5%	0.5%	8.0%
	**Orinoquía- Amazona**	8.7%	71.1%	18.6%	1.7%	20.3%
**Women**	**Atlántica**	5.2%	71.4%	21.9%	1.5%	23.4%
	**Oriental**	5.3%	64.4%	27.9%	2.4%	30.3%
	**Central**	9.1%	60.3%	28.0%	2.6%	30.6%
	**Pacífico**	3.0%	54.8%	37.6%	4.7%	42.3%
	**Bogotá**	9.5%	68.1%	20.9%	1.4%	22.4%
	**Antioquia**	14.9%	60.8%	22.5%	1.8%	24.4%
	**Valle del Cauca**	9.1%	65.1%	23.9%	1.9%	25.8%
	**San Andrés**	9.4%	79.7%	10.5%	0.4%	10.9%
	**Orinoquía- Amazona**	5.2%	68.8%	24.2%	1.8%	26.0%

All ECV-waves are pooled (2011–2019). Data includes individuals aged 24–75. Sample weights are used

#### Individual income and regional measures of income inequality

The household gross income variable is constructed by the DANE. Household gross income data is expressed in Colombian pesos (COP-$) per month and includes employment earnings (monetary and non-monetary), self-employment earnings, capital revenue, private and occupational pensions, transfer payments (e.g. subsidies), revenue arising from the sale of goods and imputed rent. Income flows of the domestic service are excluded [36]. The official consumer price index was used to adjust household income for inflation over the five years. Income figures are expressed in 2015 prices. The monthly average household income is $2,404,311 (U.S. $829), and the average per-capita household income is $585,595 (U.S. $202). Only 1,1% of observations are considered missing data and outliers. The observations with missing data (on the income variable) are distributed across the country, and are not concentrated in one particular region.

In order to account for differential needs and the existence of economies of scale in consumption within the household [37], an equivalence scale index is estimated following the methodology implemented for the construction of the Socioeconomic database for Latin America and the Caribbean (SEDLAC) [38]. The equivalence scale index is defined by the formula ${\left(A+{\alpha }_{1 }{K}_{1}+{\alpha }_{2} {K}_{2}\right)}^{\theta }$, "where A is the number of adults, K_1_ the number of children under 5 years, and K_2_ the number of children between 6 and 14. Parameters α allow for different weights for adults and kids, while θ regulates the degree of household economies of scale"[38]. As suggested by Deaton and Zaidi, the following parameter values are used: α_1_ = 0.5, α_2_ = 0.75 and θ = 0.9 [37].

Since the literature has highlighted that income inequality estimates are susceptible to the income measure that is used [39], to study the income inequality-health association, the Gini coefficient is computed for the three income measures (household income, per-capita income and equivalised-income), at regional level. The Gini-index computes the average absolute between all pairs of incomes relative to the mean and ranges from 0.0 (perfect equality) to 1.0 (perfect inequality). It is the most commonly used summary measure of income inequality and it is also relatively insensitive to income changes at the top and at the bottom of the distribution [21, 40]. Given that some studies have reported that the effect of income inequality on health is not robust to the inequality measure used [21], all estimations are repeated using three generalized entropy class inequality indices -G.E. (α): (i) the mean log deviation -G.E. (0); (ii) the Theil index -G.E. (1); and (iii) half the squared coefficient of variation -G.E. (2). The α parameter specifies the sensitivity to income differences in different parts of the income distribution; the more positive α is, the greater the sensitivity of the index to income-share differences at the upper tail [40].

**Fig. 1:**
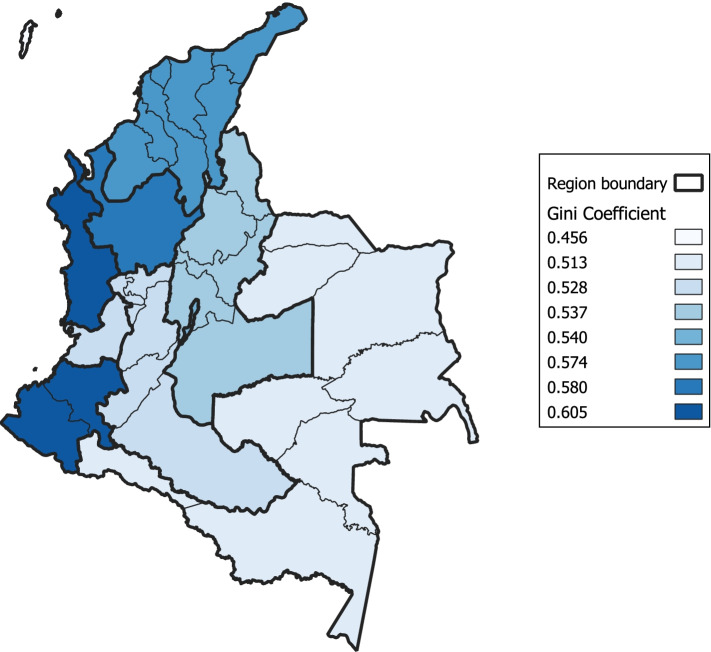
Gini-coefficient across Colombian regions. Note: Average region-level Gini-coefficients over time (2011–2019), using household income data

All income inequality indices were estimated using a Stata program developed by Jenkins (2006). Table 2 provides the different income inequality estimates. When using household income, the average Gini-coefficient is 0.51, and –on average– it ranges between 0.42 and 0.54 (see Fig. 1). Inequality tends to be greater when it is estimated with per-capita income than if estimations are performed based on household income. Inequality estimates based on per-capita income tend to be greater than those based on equivalised income; the presence of economies of scale introduces an inequality-reducing effect [41].

**Table 2:** Descriptive statistics for Aggregate Income Variables

	**Mean income in $ COP**^**a**^	**Gini**	**GE (0)**	**GE (1)**	**GE (2)**
***Region level (Sample size: 567,678)***					
Household income	2,574,604	.51	.49	.50	.97
	(935,639)	(.03)	(.06)	(.07)	(.28)
Per-capita income	735,253	.54	.55	.61	1.53
	(329,045)	(.03)	(.08)	(.09)	(.77)
Equivalised income	955,012	.52	.50	.55	1.29
	(395,919)	(.03)	(.07)	(.08)	(.56)

Observations are pooled over a 8-years period (2011–2019). Sample is limited to individuals aged between 24–75. Standard deviations are in parenthesis

^a^Weighted means are calculated using the ECV supplemental weights for individual observations

#### Control variables

The estimations performed to consider potential individual-level cofounders of any association between income inequality and self-rated health, such as age, sex, health insurance regime, education, employment, ethnicity, etc. Mean regional income and area-level socioeconomic development are added as aggregate-level control variables. Descriptive statistics for all individual covariates, including the potential individual-level cofounders, are found in Table 3.

**Table 3:** Descriptive statistics for Individual-Level Variables

	**Weighted Mean and Standard Deviation**
**Continuous Variables**	**Weighted means**
Household income ($COP 2015)	2,781,907
	(420,256)
Per-capita income ($COP 2015)	868,628
	(1,572,912)
Equivalised income ($COP 2015)	1,074,374
	(1,799,526)
Household size	3.93
	(1.92)
Age (in-years)	44
	(13.56)
	**Mean**
**Indicator Variables**	**Weighted means**
Poor or fair health status	0.23
Ethnicity/Otherwise^b^	0.11/0.89
Male/Female^b^	0.48/0.52
Married	0.23
Divorced or separated	0.10
Widowed	0.03
Singled^b^	0.64
Health insurance coverage/No insurance^b^	0.72/0.28
Health insurance regime – Contributory	0.47
Health insurance regime – Subsidized	0.42
Health insurance regime – Other/Do not Know^b^	0.11
Less than primary school^b^	0.05
Less than high school	0.38
Highschool, technical education or some college	0.32
College and advanced degree	0.10
Do not Know (Education)	0.15
Employed/Unemployed^b^	0.66/0.34
Urban/Rural^b^	0.58/0.42
Owner occupied/Rental^b^	0.52/0.48
Department level of socioeconomic development^a^	2.37

Observations are pooled over a 8-years period (2011–2019). Sample is limited to individuals aged between 24–75. Weighted means are calculated using the ECV supplemental weights for individual observations

^a^Three categories are considered: 1. Early; 2. Intermediate; 3. Advance. Educational variables are divided by the lower and upper bound, therefore Less than primary refers to a individual with no education or preschool studies, Less than high school refer to an individual who receive more than primary schooling but less than high school education, Highschool, technical education or some college refers to individuals in those cathegories and College and advanced degrees to individuals with the most education in our sample

^b^Reference category in the regression

### Empirical analysis

As documented by most ecological studies, there is a positive and significant association between aggregate-level health and income inequality in Colombia (coefficient of correlation of 0.71). Nonetheless, as stated earlier, it is essential to use individual-level data in order to disentangle the effect of individual income from the contextual effect of income inequality. Following Mellor and Milyo, the effect of income inequality on individual's health status is estimated using probit models with a pooled-cross section database [17], with our binary measure or "fair or poor" health as the dependent variable:

$${Ph}_{ijt}=\alpha +{{\beta }_{1} {Ineq}_{jt}+{\beta }_{2}X}_{ijt}+ {\beta }_{3}{MeanY}_{jt}+{y}_{t}+{e}_{ijt}$$

where ${Ph}_{ijt}$ is the dichotomized health indicator for fair/poor health status for individual *i* living in region *j* in year *t*, *Ineq*_*jt*_ is the measure of income inequality in the region of residence j at year *t*, *X*_*ijt*_ is a vector of individual-level control variables, *MeanY*_*jt*_ is the mean income of region *j* at year *t, y*_*t*_ is a year-dummy and ${e}_{ijt}$ the error term. As suggested by the same authors, a spline function is estimated over individual income –with the knots defined at the quintiles of the distribution of income- in order to allow for nonlinearity in the relationship between health and individual income [17]. Even though within-region income inequality is rather stable over time, there is considerable variation across regions. Hence, to take advantage of such variability, regional fixed effects are not considered in the model specification, meaning that we are not holding constant any time-invariant within-region factors. To account for potential unobserved heterogeneity associated with area-level determinants of individual health (e.g. patterns of health care suppliers), a categorical variable that establishes the level of department socioeconomic development is included in the estimations. Colombia is divided into 32 departments and a Capital district, and the ECV specifies the department of residence of each individual from the sample. This categorical variable was developed by the National Planning Department to make resource allocation decisions [42].

## Results

### Strong IIH

Table 4 reports the estimated average marginal effects (AMEs) with household income as the individual-level income measure. Household income was used for estimating inequality measures, income quintiles, and regional average income. When using the Gini coefficient, the estimated marginal effect of regional inequality on health status is positive but not statistically significant (Column 1). When control variables are added, the magnitude of the effect is greater and is statically significant (*p* < 0.01) (Column 2). The magnitude of the marginal effect of income inequality on health is smaller when using the generalized entropy measures (GE (0) and G(1)) but remains statically significant even after controlling for individual and area-level characteristics (Columns 4 and 6).

**Table 4:** Effect of Income Inequality on Individual Health Status: Average Marginal Effects from Probit Models Reported

	**Dependent Variable: 1 if "Fair" or "Poor" reported health status**							
**Inequality measure:**	**Gini coefficient**		**GE (0)**		**GE (1)**		**GE (2)**	
	**(1)**	**(2)**	**(3)**	**(4)**	**(5)**	**(6)**	**(7)**	**(8)**
Inequality	0.33	0.54^a^	0.12	0.30^a^	0.12	0.18^b^	0.014	0.022
	(0.67)	(3.00)	(0.50)	(3.08)	(0.62)	(2.44)	(0.38)	(1.14)
Region Mean Income	-0.038^b^	-0.051^a^	-0.039^c^	-0.048^a^	-0.039^c^	-0.053^a^	-0.041^c^	-0.057^a^
	(1.96)	(4.02)	(1.93)	(3.54)	(1.90)	(4.02)	(1.76)	(3.88)
Household income								
Q1		-0.082^a^		-0.081^a^		-0.083^a^		-0.085^a^
		(5.23)		(5.21)		(5.26)		(5.35)
Q2		-0.081^a^		-0.082^a^		-0.082^a^		-0.085^a^
		(6.68)		(6.58)		(6.74)		(6.71)
Q3		-0.006		-0.006		-0.006		-0.006
		(0.75)		(0.74)		(0.76)		(0.78)
Q4		-0.036^a^		-0.036^a^		-0.036^a^		-0.036^a^
		(7.95)		(7.86)		(7.90)		(7.83)
Q5		-0.003^a^		-0.003^a^		-0.003^a^		-0.003^a^
		(4.94)		(4.95)		(4.94)		(4.87)
Age/100		1.05^a^		1.05^a^		1.05^a^		1.04^b^
		(21.19)		(21.45)		(21.16)		(21.02)
(Age/100)^2^		-0.30^a^		-0.30^a^		-0.30^a^		-0.30^a^
		(6.69)		(6.67)		(6.63)		(6.52)
Household size		0.005^a^		0.005^a^		0.005^a^		0.005^a^
		(4.73)		(4.90)		(4.83)		(4.95)
Male		-0.066^a^		-0.066^a^		-0.067^a^		-0.067^a^
		(21.61)		(21.46)		(21.47)		(21.32)
Ethnicity		0.011		0.012		0.011		0.013^c^
		(1.38)		(1.51)		(1.36)		(1.65)
Married		0.002		0.001		0.002		0.002
		(0.38)		(0.30)		(0.40)		(0.38)
Widowed		-0.002		-0.002		-0.002		-0.002
		(0.24)		(0.31)		(0.25)		(0.28)
Contributory regime		-0.028^a^		-0.028^a^		-0.028^a^		-0.028^a^
		(6.90)		(6.98)		(6.93)		(6.86)
Subsidized regime		0.019^a^		0.019^a^		0.020^a^		0.020^a^
		(5.10)		(5.06)		(5.24)		(5.54)
Less high school		0.003		0.003		0.003		0.003
		(0.55)		(0.52)		(0.55)		(0.54)
Technical education or some college		-0.066^a^		-0.066^a^		-0.066^a^		-0.066^a^
		(9.10)		(9.20)		(9.06)		(9.07)
College or advanced degree		-0.12^a^		-0.12^a^		-0.12^a^		-0.12
		(13.25)		(13.37)		(12.97)		(12.80)
Employed		-0.056^a^		-0.055^a^		-0.055^a^		-0.055^a^
		(20.35)		(19.90)		(20.12)		(19.50)
Urban		0.0005		0.0006		0.0001		0.0003
		(0.01)		(0.13)		(0.03)		(0.08)
Owner-occupied		0.003		0.003		0.004		0.004
		(1.13)		(1.18)		(1.28)		(1.53)
Household head		0.007^a^		0.007^a^		0.007^a^		0.007^a^
		(2.99)		(2.97)		(3.01)		(3.01)
Plus department socio-economic development	No	Yes	No	Yes	No	Yes	No	Yes
Wald chi-squared	737	32,481		33,081		34,012		36,375
*P*-value	0.000	0.000		0.000		0.000		0.000
Pseudo R2	0.024	0.119	0.008	0.120	0.008	0.120	0.008	0.119

Absolute t-statistics are reported in parentheses. ^a^ denotes significance at 1%, ^b^ at 5%, and ^c^ at 10%. All ordered-probit models were estimated with standard errors adjusted for clustering at regional level. Since Colombia has a universal health insurance scheme, dummies for the most prominent health insurance regimes (contributory and subsidized) were included instead of a general-health insurance indicator. All estimations include year dummies

Region-level mean income is also statically significant but negative, and remains as such after including control variables. This suggests that living in regions with higher mean income reduces the likelihood of reporting fair/poor health. Across all estimations -regardless of the income inequality measure used- the marginal effect of household income is negative and statically significant at each income quintile, except among the third quintile. An income gradient across the first, second and third quintiles was observed in all estimations, with the association between household income and the probability of reporting fair/poor health being larger among the poorest 20%. These findings partially support the absolute income hypothesis, which posits that individual's health improves with increases in individual living standards [43].

Results related to the impact of the individual characteristics on individuals’ health status are in line with what was expected and with findings from previous studies. Respondents with a contributory health regime (i.e. individuals who financially contribute to the health system) are on average 2.8 percentage points (pp) less likely to report being in fair or poor health, while those who belong to the subsidized regime (i.e. poor and vulnerable population) are just under 2.0 pp more likely to report fair/poor health status. These results are not surprising since individuals under the subsidized regime tend to experience low living-standards. Similar to most studies, the lower the education level, the greater the probability of reporting poor or fair health status. Being employed has a significant negative correlation with the probability of reporting fair/poor health, while having a big household size increases the chances of being in poor or fair health. Unlike most U.S. studies, the ethnicity variable (i.e. being indigenous, gypsy, raizal, afro or palenquero) seems irrelevant for the Colombian setting.

Estimations using per-capita income and equivalised income yield similar results (See Additional file 1, Table 1). The marginal effect of income inequality is positive and statically significant when using the Gini coefficient. As in Table 4, even though the magnitude of the marginal effect is lessened when using the generalized entropy indices, it remains statically significant.

In summary, there is a strong statistical association between regional income inequality and self-rated health. Such association appears to be robust to the choice of income inequality measure, although the magnitude of the marginal effect is considerably smaller when using generalised entropy measures.

### Weak IIH

To test whether income inequality particularly affects the health of the poor, we allow the effect of income inequality to vary by the individual's income level by including in the estimations five dummy variables based on quintiles in the distribution of individual's income. In other words, binary variables are created for each income quintile, and we interact each one of them with the income inequality measure.

Table 5 reports estimations for the four indices of income inequality considered when household income is used as the individual-income measure. When inequality is operationalized with the Gini coefficient and controls are added (Column 2), at any level of household income, the coefficients of the interaction terms are positive, and the effect is statically significant (*p* < 0.01). Similar patterns of results were obtained when G.E. (0) and G.E. (1) were used to calculate income inequality (Columns 4 and 6), although the magnitude of the marginal effect is considerably attenuated. Similar results are found when using per-capita income and equivalised income.

**Table 5:** Effect of Income Inequality on Individual Health Status: Average Marginal Effects from Probit Models Reported

	**Dependent Variable: 1 if "Fair" or "Poor" reported health status**							
**Inequality measure:**	**Gini coefficient**		**GE(0)**		**GE(1)**		**GE(2)**	
	**(1)**	**(2)**	**(3)**	**(4)**	**(5)**	**(6)**	**(7)**	**(8)**
Inequality								
Q1	0.38	0.54^a^	0.22	0.32^a^	0.23^b^	0.18^b^	0.073^a^	0.020
	(1.50)	(2.92)	(1.59)	(3.14)	(2.28)	(2.22)	(2.98)	(0.85)
Q2	0.24	0.52^a^	0.082	0.29^a^	0.095	0.16^b^	0.011	0.014
	(0.98)	(2.88)	(0.62)	(2.98)	(0.96)	(2.11)	(0.45)	(0.72)
Q3	0.19	0.54^a^	0.031	0.29^a^	0.046	0.18^b^	-0.013	0.021
	(0.77)	(3.04)	(0.23)	(3.00)	(0.46)	(2.50)	(0.58)	(1.21)
Q4	0.13	0.58^a^	-0.035	0.31^a^	-0.018	0.22^a^	-0.044^c^	0.043^b^
	(0.52)	(3.28)	(0.26)	(3.24)	(0.18)	(3.15)	(1.89)	(2.39)
Q5	0.023	0.53^a^	-0.14	0.26^a^	-0.12	0.17^b^	-0.095^a^	0.026
	(0.09)	(3.00)	(1.04)	(2.71)	(1.23)	(2.51)	(3.97)	(1.53)
Region Mean Income	-0.017	-0.051^a^	-0.018^c^	-0.048^a^	-0.018	-0.053^a^	-0.021^c^	-0.057^a^
	(1.53)	(4.00)	(1.65)	(3.54)	(1.56)	(4.00)	(1.75)	(3.86)
Household income								
Q1		-0.083^a^		-0.082^a^		-0.083^a^		-0.085^a^
		(5.21)		(5.13)		(5.22)		(5.34)
Q2		-0.057^a^		-0.048^b^		-0.056^b^		-0.067^a^
		(2.71)		(2.34)		(2.67)		(2.99)
Q3		-0.030		-0.016		-0.031		-0.026
		(1.59)		(0.77)		(1.51)		(1.59)
Q4		-0.041^a^		-0.032^a^		-0.044^a^		-0.047^a^
		(4.49)		(3.52)		(4.77)		(6.23)
Q5		-0.002^a^		-0.002^a^		-0.002^a^		-0.002^a^
		(2.74)		(2.62)		(2.85)		(3.13)
Wald chi-squared	621	72,430	555	73,752	560	70,832	429	70,664
*P* value	0.000	0.000	0.000	0.000	0.000	0.000	0.000	0.000
Pseudo R2	0.024	0.120	0.023	0.120	0.023	0.120	0.022	0.119

Absolute t-statistics are reported in parentheses. ^a^ denotes significance at 1%, ^b^ at 5%, and ^c^ at 10%. In all regression-models we use clustering of standard errors at regional level. All estimations include year dummies. Estimations (2), (4), (6) and (8) include individual characteristics and a categorical variable that indicates the level of socio-economic development of the department of residence

Regardless of the differences in terms of statistical significance across the different estimations, in every specification that controls for potential cofounders, the magnitude of the marginal effect of income inequality is very similar between income quintiles. In fact, when testing whether coefficients of income inequality across income quintiles are the same, we were unable to reject the equality hypothesis at conventional levels (i.e. null hypothesis of a common coefficient). These results are not consistent with the weak version of the IIH.

All in all, we fail to find evidence in favour of the weak version of the IIH. The income-inequality—health association is not simply circumscribed to individuals with low-living standards, but instead, it seems to be present across all income quintiles, and the magnitude of the effect seems to be uniform across socioeconomic strata. When using either per-capita or equivalised income, there are some differences in terms of statistical significance of the interaction terms, which are probably reflecting the presence of greater noise (See Additional file 1, Table 2). Nevertheless, the null hypothesis of a common coefficient cannot be rejected.

### Robustness check

To study the robustness of the results obtained above, estimations for reduced samples of the original ECV sample are run. Firstly, all estimations are repeated for urban areas only (See Additional file 1, Tables 3.1 and 3.2). On average, it appears that in urban settings fewer individuals are inclined to report fair/poor health status in comparison to individuals living in hamlets or rural areas. The patterns of the results are the same as the ones found in the previous section; the marginal effect of regional income inequality is statically significant after controlling for potential cofounders, and, at any level of household income, the marginal effect of income inequality on individual's health is statically significant across all estimations.

Following the methodology of the authors Mellor and Milyo, the sample is restricted to household heads (See Additional file 1, Tables 4.1 and 4.2) [17]. As stated by the authors, income inequality is expected to have a greater impact on individuals who run their households. Once again, the marginal effect of income inequality is statically significant when using the Gini coefficient (< 0.01). The effect is diminished when using the generalized entropy indices but remains statically significant even after adding control variables. Regarding the weak IIH, the patterns observed are akin to the ones found in the previous section.

We also considered the possibility of a non-linear relationship between income inequality and health self-status, by using a restricted cubic spline on the inequality measures used. When using Gini and GE(1) there is evidence of a non-linear relationship, suggesting the existence of a threshold above which income inequality has a greater effect on individual's health (See Additional file 2, Fig. 2).

Finally, it is important to sign that the ECV imputed-rent component of household income is obtained from asking individuals directly to estimate the rent they would pay if they had to rent the dwellings they occupy. This self-assessment approach is not as robust as techniques based on econometric estimations (e.g. opportunity cost approach), and the methodological approach used for calculating imputed-rent is relevant for the results on the impact of imputed-rent on income inequality and poverty [44]. When using the self-assessment approach, one could argue issues of reliability in the answers provided by the interviewees, especially in areas where the housing market is not well developed [45]. In Colombia, this can potentially be a problem in non-urban areas. Estimations are therefore repeated with a measure of household income that excludes the ECV imputed rent component and increases the household income of housing-owners by 10% (See Additional file 43, Tables 5.1 and 5.2). This latter adjustment is implemented by SEDLAC when self-assessment of imputed rent provided by respondents are unreliable. Estimations for the strong IIH and the weak IIH are similar to the ones obtained in the previous Sect. [45].

## Discussion

After controlling for individual's income, average regional income, and other potential cofounders, estimations point to a robust statistical association between income inequality and self-rated health in Colombia. These results are robust to the income-unit of analysis and changes in the sample. The marginal effect is larger in urban settings. In addition, evidence in favour of the weak version of the IIH was not found. When using the Gini coefficient, income differences seem to have adverse effects for individuals at any level of household income. The magnitude of the marginal effect of income inequality on individual's health does not vary much between income-quintiles, suggesting that the association between regional income inequality and individual's heath extends across all socioeconomic strata. These results are also supportive of the findings in favour of the strong IIH. These findings are consistent with those of the literature [18, 25], who also observed an independent association between high levels of income inequality and a greater probability of reporting fair/poor health within the U.S. and Chile, respectively.

The relevance of assessing the sensitivity of results to the choice of income inequality measure has been highlighted by numerous studies [8, 21], and is also evident in this paper; for the Colombian context, it was found that the association between income inequality on self-reported health tends to be weaker when income inequality is operationalized with generalized entropy indices with relatively greater sensitivity to income differences at the upper tail of the distribution (i.e. G.E. (α > 0)), than when using G.E. (α <  = 0) or the Gini coefficient. Given that all inequality indices weigh incomes and income changes at different parts of the distribution differently, and the various measures of inequality might have different influences on health, it is pertinent always to examine the robustness of the income inequality—health association with a variety of inequality measures [46]. This study finds that the measure of income used in the analysis is also relevant. Even though the differences in the results are not striking, the marginal effect of income inequality is smaller when using per-capita income across all estimations. This is not entirely surprising since different income inequality estimations were obtained depending on the income-unit of analysis used. These results contrast with the findings of Kawachi and Kennedy, who found no differences in the income inequality/mortality association after adjusting for taxes, transfers, and household size (using equivalence scales) [47].

The findings of this study are consistent with the "threshold hypothesis" [4, 9], which posits that there is a threshold above which income inequality affects health outcomes; therefore, it is more likely to find support to the IIH in countries with –relatively- more unequal income distribution than the U.S. As stated in the introduction, income is more unequally distributed in Colombia than in the U.S. Future research could test the "threshold effect" of income inequality on fair/poor self-rated health [25], and the pathways that might explain this association throughout the income distribution. Both exceed the scope of the present study.

In terms of policy implications, in the light of the results obtained, it is crucial for the Colombian government to strengthen its efforts to reduce disparities across socioeconomic groups. Income inequality has always been a serious issue in Colombia [48], and its impact on health takes on greater relevance in today's time due to COVID-19 pandemic, first identified in December 2019. Various studies have pointed out the devastating impact of this virus on poverty and inequality across Latin America, with the number of poor people rising in more than 200 million in 2020, and the average Gini index estimated to be 2.9% higher than the 2019 estimate [49].

The results of this study reinforce the recommendations stated in the Adelaide Statement [50]: Health must be present in all policies. Given that the causes of health are socially and economically originated, the responsibility of improving people's health is not confined to the health sector but to all governmental institutions in charge of improving society's living standards and reducing socioeconomic disparities.

Finally, a couple of limitations merit comment. First, we used household income data unadjusted for taxation and social insurance contributions. It can be argued that net (or disposable) household income is a better –more comprehensive- measure of living standards. Considering these other components into the income variable can make a difference to distributional assessments and might affect the association found between income inequality and self-rated health. It is also important to consider the presence of measurement error in the gross household income variable used. Even though, traditionally, consumption has been considered a more appropriate measure for distributional analysis, reporting issues have favoured the use of income data [46, 51]. Nevertheless, in developing countries, measurement error in consumption tends to be less pronounced; in other words, in poor families, income data tends to be substantially under-reported and have higher non-response rates than consumption data [51, 52]. Therefore, replicating the analyses performed in this paper with consumption expenditure data or with another proxy variable of socioeconomic status is a suggested step to follow. Up to date, however, there is no consumption data on a yearly basis for Colombia. Most studies that explore the association between inequality and health have focused on income inequality, and relatively few have examined the validity of the IIH by using alternative measures of social status. Income-based inequality is just one dimension of inequality, and other social hierarchy markers (e.g. distribution of wealth) can potentially impact –even greater than income inequality- on an individual's health [4].

Second, the reliance on one health outcome measure might casts doubts upon the reliability of the results. The 4-scale self-rated health status measure is not likely to capture the complexity and diversity of individuals' health; it is therefore pertinent to incorporate various health indicators in any health analysis [53]. On the other hand, given that self-reported health could reflect individuals' perceptions or expectations, it is common to find that low-income individuals tend to report poor health status in developing countries because of their lower expectations of health [54]. Nevertheless, within Colombia, we found that among the poor, a smaller proportion of individuals report having a good/very good health status than among the rich. It could also be argued that measures of self-reported health cannot be entirely interpreted comparably across regions because of implicit differences in norms, values and health-expectations [22]. Although this concern has primarily been of capital importance in cross-country studies, it has been suggested that even within-country studies -in large nations (eg. The U.S.)- could be affected by norms and expectations differences across states [22]. However, across Colombian regions, it is not likely to find significant differences –in terms of norms and expectations- that affect the comparability of self-reported health data; Colombia is significantly smaller than the U.S., and even though there are some cultural differences, the healthcare system is the same for the whole country.

Third, income distribution measures were calculated at the regional level since there was no data available to calculate income inequality at a lower disaggregation level (e.g. departments). Apart from the potentially few variations with a nine regions sample, it is worth noticing that social policy decisions are mainly implemented at a department level. If the association between income inequality and fair/poor health status is –at least partly- driven by the "*policy pathway*", which posits that income disparities affect health negatively through the implementation of certain social and health policies that do not benefit the poor [55–58], an analysis based on department-level income inequalities is likely to be more accurate. The geographic scale at which income inequality is estimated has been subject to debate. Within the U.S., studies that estimate income inequality at the state level have found evidence in favour of the IIH, while studies at lower levels of aggregation have been less supportive of an association between income inequality and health [4]; "the state-level associations seem to suggest the importance of political mechanisms, such as the relation of economic disparities within each state to patterns of spending by state legislatures on social goods such as health care, education, and welfare" [4]. Such findings have been supported by evidence from the European Union [22].

The fourth limitation has to do with the difficulty of concluding a causal link between income inequality and self-rated health, given that this study was based on pooled-cross sectional data. The possibility of a bi-directional relationship between both variables cannot be ruled out (i.e. a large proportion of unhealthy people increases income inequality). However, some time-series and panel studies have disapproved this possibility [10]. Finally, it is difficult to exclude the possibility of an omitted variable bias; some area-level characteristics -not included in the estimation- might be driving the association found between income inequality and self-rated health.

## Conclusions

We explored the association between regional income inequality and individual's self-rated health status in Colombia, using the ECV household survey (2011–2019). After controlling for individual income levels, regional average income and socioeconomic characteristics, evidence of an independent association between regional-income inequality and the probability of reporting fair/poor health were found. Such association is not only confined to low-income individuals but extends across all socioeconomic strata. This association is robust to the income inequality measure implemented, the income-unit of analysis, and changes in the sample. Nevertheless, a weaker marginal effect of income inequality on health was identified when using generalized entropy indices with relatively greater sensitivity to income gaps among the rich or when using per-capita income in the analyses. This seems to be consistent with the fact that the effect of income inequality on individual’s self-rated health status is stronger among the poor.

This is the first study that examines the validity of the IIH within Colombia and is one of the few in middle-income economies. Although some limitations were acknowledged, this study is considered to contribute to the literature of this nature, particularly given the limited number of studies focused on developing or emerging economies. Further research studies that test the IIH in relatively high unequal societies are, however, needed.

It is suggested that reducing income disparities can potentially contribute to improving individual's health. The Colombian government should put more efforts into reducing living-standards disparities, not only for the sake of individual's health but for the sake of social cohesion and democracy; "of all the costs imposed on our society by the top 1 percent, perhaps the greatest is this: the erosion of our sense of identity in which fair play, equality of opportunity, and a sense of community are so important" (Stiglitz, 2015:93).

## Supplementary Information

**Additional file 1: Table 1.** Effect of Income Inequality on Individual Health Status: Average Marginal Effects from Probit Models Reported (Using (A= Per-capita income or (B) Equivalised income). **Table 2.** Effect of Income Inequality on Individual Health Status: Average Marginal Effects from Probit Models Reported (Using (A) Per-capita income or (B) Equivalised income). **Table 3.1.** Effect of Income Inequality on Individual Health Status: Average Marginal Effects from Probit Models (Urban population). **Table 3.2.** Effect of Income Inequality on Individual Health Status: Average Marginal Effects from Probit Models (Urban population). **Table 4.1.** Effect of Income Inequality on Individual Health Status: Average Marginal Effects from Probit Models (Household heads). **Table 4.2.** Effect of Income Inequality on Individual Health Status: Average Marginal Effects from Probit Models (Household heads). **Table 5.1.** Effect of Income Inequality on Individual Health Status: Average Marginal Effects from Probit Models (Household income without imputed rent and adjusted following SEDLAC). **Table 5.2.** Effect of Income Inequality on Individual Health Status: Average Marginal Effects from Probit Models (Household income without imputed rent and adjusted following SEDLAC).

**Additional file 2: Table 6.** Effect of Income Inequality on Individual Health Status: Average Marginal Effects from the Probit Multilevel Regressions. **Table 7.** Effect of Income Inequality on Chronic Heart Disease Status: Average Marginal Effects from Probit Models. **Figure 2**. Non-linear strong IIH test using restricted cubid splines.

## Data Availability

The datasets analysed during the current study are publicly available at https://www.dane.gov.co/.
