# Supplementary material for: Income inequality and self-rated health status in Colombia
Source: Int J Equity Health. 2022 May 16;21:69. doi: 10.1186/s12939-022-01659-8 (PMC9108691; doi:10.1186/s12939-022-01659-8)
Supplement: Supplementary file 1 — Additional file 1: Table 1. Effect of Income Inequality on Individual Health Status: Average Marginal Effects from Probit Models Reported (Using (A= Per-capita income or (B) Equivalised income). Table 2. Effect of Income Inequality on Individual Health Status: Average Marginal Effects from Probit Models Reported (Using (A) Per-capita income or (B) Equivalised income). Table 3.1. Effect of Income Inequality on Individual Health Status: Average Marginal Effects from Probit Models (Urban population). Table 3.2. Effect of Income Inequality on Individual Health Status: Average Marginal Effects from Probit Models (Urban population). Table 4.1. Effect of Income Inequality on Individual Health Status: Average Marginal Effects from Probit Models (Household heads). Table 4.2. Effect of Income Inequality on Individual Health Status: Average Marginal Effects from Probit Models (Household heads). Table 5.1. Effect of Income Inequality on Individual Health Status: Average Marginal Effects from Probit Models (Household income without imputed rent and adjusted following SEDLAC). Table 5.2. Effect of Income Inequality on Individual Health Status: Average Marginal Effects from Probit Models (Household income without imputed rent and adjusted following SEDLAC). [file 12939_2022_1659_MOESM1_ESM.docx]

**Supplemental material – Additional File 1**

**Table 1. Effect of Income Inequality on Individual Health Status: Average Marginal Effects from Probit Models Reported (Using (A= Per-capita income or (B) Equivalised income)**

|  | **Dependent Variable: 1 if "Fair" or "Poor" reported health status** | | | | | | | | |
| --- | --- | --- | --- | --- | --- | --- | --- | --- | --- |
| **Inequality measure:** | **Gini coefficient** | | **GE (0)** | | **GE (1)** | | **GE (2)** | | |
| **(A)** | (1) | (2) | (3) | (4) | (5) | (6) | (7) | (8) |  |
| Inequality | 0.26 | 0.43** | 0.067 | 0.22** | 0.072 | 0.098 | 0.010 | 0.0130** |  |
|  | (0.54) | (2.28) | (0.32) | (2.48) | (0.42) | (1.41) | (0.88) | (2.24) |  |
| Region Mean Income | -0.087 | -0.12*** | -0.092 | -0.11*** | -0.088 | -0.12*** | -0.087 | -0.12*** |  |
|  | (1.52) | (3.12) | (1.50) | (2.82) | (1.63) | (3.09) | (1.39) | (2.77) |  |
| Per-capita income |  |  |  |  |  |  |  |  |  |
| Q1 |  | -0.16*** |  | -0.15*** |  | -0.16*** |  | -0.16*** |  |
|  |  | (2.80) |  | (2.77) |  | (2.88) |  | (2.96) |  |
| Q2 |  | -0.22*** |  | -0.22*** |  | -0.22*** |  | -0.22*** |  |
|  |  | (5.46) |  | (5.41) |  | (5.54) |  | (5.33) |  |
| Q3 |  | -0.11*** |  | -0.11*** |  | -0.11*** |  | -0.11*** |  |
|  |  | (4.19) |  | (4.12) |  | (4.26) |  | (4.30) |  |
| Q4 |  | -0.15*** |  | -0.15*** |  | -0.15*** |  | -0.15*** |  |
|  |  | (10.74) |  | (10.65) |  | (10.79) |  | (10.91) |  |
| Q5 |  | -0.009*** |  | -0.009*** |  | -0.009*** |  | -0.009*** |  |
|  |  | (4.20) |  | (4.20) |  | (4.16) |  | (4.07) |  |
| Wald chi-squared | . | 37,130 | . | 36,092 | . | 40,801 | . | 43,017 |  |
| P value | . | 0.000 | . | 0.000 | . | 0.000 | . | 0.000 |  |
| Pseudo R2 | 0.007 | 0.119 | 0.007 | 0.119 | 0.007 | 0.119 | 0.0067 | 0.119 |  |

| **(B)** | (1) | (2) | (3) | (4) | (5) | (6) | (7) | (8) |
| --- | --- | --- | --- | --- | --- | --- | --- | --- |
| Inequality | 0.32 | 0.48*** | 0.11 | 0.26*** | 0.099 | 0.12* | 0.014 | 0.017* |
|  | (0.69) | (2.63) | (0.48) | (2.79) | (0.56) | (1.70) | (0.83) | (1.70) |
| Region Mean Income | -0.075 | -0.096*** | -0.078 | -0.092*** | -0.076 | -0.10*** | -0.076 | -0.10*** |
|  | (1.55) | (3.16) | (1.52) | (2.75) | (1.60) | (3.12) | (1.47) | (2.82) |
| Equivalised income |  |  |  |  |  |  |  |  |
| Q1 |  | -0.16*** |  | -0.16*** |  | -0.16*** |  | -0.16*** |
|  |  | (3.78) |  | (3.77) |  | (3.86) |  | (3.94) |
| Q2 |  | -0.15*** |  | -0.15*** |  | -0.15*** |  | -0.16*** |
|  |  | (4.42) |  | (4.40) |  | (4.49) |  | (4.41) |
| Q3 |  | -0.083*** |  | -0.082*** |  | -0.084*** |  | -0.085*** |
|  |  | (3.71) |  | (3.66) |  | (3.78) |  | (3.84) |
| Q4 |  | -0.11*** |  | -0.11*** |  | -0.11*** |  | -0.11*** |
|  |  | (9.27) |  | (9.16) |  | (9.23) |  | (9.30) |
| Q5 |  | -0.008*** |  | -0.008*** |  | -0.008*** |  | -0.008*** |
|  |  | (54.38) |  | (4.39) |  | (4.34) |  | (4.25) |
| Wald chi-squared | . | 31,226 | . | 33,501 | . | 33,407 | . | 33,799 |
| P value | . | 0.000 | . | 0.000 | . | 0.000 | . | 0.000 |
| Pseudo R2 | 0.007 | 0.119 | 0.007 | 0.119 | 0.007 | 0.118 | 0.007 | 0.119 |

Note. Absolute t-statistics are reported in parentheses. *** denotes significance at 1%, ** at 5%, and * at 10%. In all regression models we use clustering of standard errors. All estimations include year dummies. Estimations (2), (4) and (6) of table A and B include individual characteristics and a categorical variable that indicates the level of socio-economic development of the department of residence. In table A, inequality measures, income quintiles and average regional income were estimated using per-capita income. Similarly, in table B, equivalised income was used in all estimations.

**Table 2. Effect of Income Inequality on Individual Health Status: Average Marginal Effects from Probit Models Reported (Using (A) Per-capita income or (B) Equivalised income)**

|  | **Dependent Variable: 1 if "Fair" or "Poor" reported health status** | | | | | | | |
| --- | --- | --- | --- | --- | --- | --- | --- | --- |
| **Inequality measure:** | **Gini coefficient** | | **GE (0)** | | **GE (1)** | | **GE (2)** | |
| **(A)** | (1) | (2) | (3) | (4) | (5) | (6) | (7) | (8) |
| Inequality |  |  |  |  |  |  |  |  |
| Q1 | 0.27 | 0.45** | 0.13 | 0.24** | 0.13 | 0.10 | 0.029*** | 0.013** |
|  | (1.17) | (2.27) | (1.16) | (2.51) | (1.56) | (1.32) | (3.70) | (2.03) |
| Q2 | 0.21 | 0.43** | 0.074 | 0.22** | 0.077 | 0.091 | 0.015* | 0.013* |
|  | (0.92) | (2.22) | (0.67) | (2.40) | (0.94) | (1.24) | (1.71) | (1.96) |
| Q3 | 0.16 | 0.43** | 0.023 | 0.21** | 0.032 | 0.091 | -0.001 | 0.010 |
|  | (0.70) | (2.29) | (0.21) | (2.45) | (0.40) | (1.37) | (0.09) | (1.56) |
| Q4 | 0.087 | 0.44** | -0.050 | 0.21*** | -0.033 | 0.11* | -0.023* | 0.019*** |
|  | (0.38) | (2.40) | (0.45) | (2.60) | (0.42) | (1.85) | (1.95) | (3.82) |
| Q5 | -0.019 | 0.41** | -0.15 | 0.18** | -0.13 | 0.094 | -0.056*** | 0.015*** |
|  | (0.08) | (2.23) | (1.36) | (2.16) | (1.56) | (1.55) | (3.57) | (2.82) |
| Region Mean Income | -0.028 | -0.12*** | -0.034 | -0.11*** | -0.032 | -0.12*** | -0.051 | -0.12*** |
|  | (0.92) | (3.11) | (1.10) | (2.82) | (1.05) | (3.09) | (1.47) | (2.75) |
| Per-capita income |  |  |  |  |  |  |  |  |
| Q1 |  | -0.15** |  | -0.14** |  | -0.15*** |  | -0.17*** |
|  |  | (2.65) |  | (2.53) |  | (2.79) |  | (3.05) |
| Q2 |  | -0.13* |  | -0.11 |  | -0.16* |  | -0.22*** |
|  |  | (1.89) |  | (1.36) |  | (1.91) |  | (3.39) |
| Q3 |  | -0.13* |  | -0.10 |  | -0.14** |  | -0.099** |
|  |  | (2.01) |  | (1.48) |  | (2.17) |  | (2.62) |
| Q4 |  | -0.14*** |  | -0.13*** |  | -0.16*** |  | -0.18*** |
|  |  | (5.35) |  | (4.93) |  | (5.63) |  | (9.26) |
| Q5 |  | -0.007*** |  | -0.007*** |  | -0.007*** |  | -0.008*** |
|  |  | (3.52) |  | (3.46) |  | (3.56) |  | (3.65) |
| Wald chi-squared | 482 | 63,091 | 433 | 76,518 | 428 | 73,790 | 162. | 50,548 |
| P value | 0.000 | 0.000 | 0.000 | 0.000 | 0.000 | 0.000 | 0.000 | 0.000 |
| Pseudo R2 | 0.019 | 0.119 | 0.018 | 0.119 | 0.018 | 0.119 | 0.016 | 0.119 |

| (B) | (1) | (2) | (3) | (4) | (5) | (6) | (7) | (8) |
| --- | --- | --- | --- | --- | --- | --- | --- | --- |
| Inequality |  |  |  |  |  |  |  |  |
| Q1 | 0.35 | 0.48*** | 0.19 | 0.27*** | 0.17** | 0.12 | 0.042*** | 0.016 |
|  | (1.53) | (2.59) | (1.61) | (2.80) | (1.99) | (1.48) | (3.41) | (1.40) |
| Q2 | 0.26 | 0.48** | 0.11 | 0.26*** | 0.099 | 0.11 | 0.017 | 0.016 |
|  | (1.16) | (2.55) | (0.93) | (2.70) | (1.12) | (1.47) | (1.29) | (1.48) |
| Q3 | 0.21 | 0.50*** | 0.049 | 0.27*** | 0.046 | 0.14* | -0.004 | 0.017* |
|  | (0.91) | (2.72) | (0.42) | (2.88) | (0.52) | (1.91) | (0.26) | (1.78) |
| Q4 | 0.12 | 0.50*** | -0.035 | 0.26*** | -0.031 | 0.15** | -0.033** | 0.027*** |
|  | (0.55) | (2.79) | (0.30) | (2.93) | (0.36) | (2.26) | (2.22) | (3.08) |
| Q5 | 0.012 | 0.45** | -0.15 | 0.21** | -0.13 | 0.11 | -0.072*** | 0.018* |
|  | (0.05) | (2.50) | (1.24) | (2.28) | (1.53) | (1.68) | (4.07) | (1.96) |
| Region Mean Income | 0.021 | 0.096*** | -0.025 | -0.092*** | -0.024 | -0.10*** | -0.040 | -0.10*** |
|  | (0.83) | (3.14) | (0.97) | (2.75) | (0.93) | (3.11) | (1.36) | (2.80) |
| Equivalised income |  |  |  |  |  |  |  |  |
| Q1 |  | -0.16*** |  | -0.15*** |  | -0.16*** |  | -0.16*** |
|  |  | (3.68) |  | (3.61) |  | (3.82) |  | (3.98) |
| Q2 |  | -0.12** |  | -0.11* |  | -0.14** |  | -0.15*** |
|  |  | (2.47) |  | (1.90) |  | (2.36) |  | (2.83) |
| Q3 |  | -0.16*** |  | -0.128*** |  | -0.17*** |  | -0.11*** |
|  |  | (3.63) |  | (2.59) |  | (3.45) |  | (3.00) |
| Q4 |  | -0.074*** |  | -0.063*** |  | -0.094*** |  | -0.12*** |
|  |  | (3.34) |  | (2.89) |  | (4.05) |  | (7.58) |
| Q5 |  | -0.006*** |  | -0.006*** |  | -0.006*** |  | -0.007*** |
|  |  | (3.27) |  | (3.17) |  | (3.31) |  | (3.55) |
| Wald chi-squared | 496 | 40,807 | 451 | 46,881 | 446 | 37,618 | 231 | 46,263 |
| P value | 0.000 | 0.000 | 0.000 | 0.000 | 0.000 | 0.000 | 0.000 | 0.000 |
| Pseudo R2 | 0.021 | 0.119 | 0.021 | 0.119 | 0.021 | 0.119 | 0.019 | 0.119 |

Note. Absolute t-statistics are reported in parentheses. *** denotes significance at 1%, ** at 5%, and * at 10%. In all regression models we use clustering of standard errors. All estimations include year dummies. Estimations (2), (4), (6) and (8) include individual characteristics and a categorical variable that indicates the level of socio-economic development of the department of residence. In table A, inequality measures, income quintiles and average regional income were estimated using per-capita income. Similarly, in table B, equivalised income was used in all estimations.

**Table 3.1. Effect of Income Inequality on Individual Health Status: Average Marginal Effects from Probit Models (Urban population)**

|  | **Dependent Variable: 1 if "Fair" or "Poor" reported health status** | | | | | | | |
| --- | --- | --- | --- | --- | --- | --- | --- | --- |
| **Inequality measure:** | **Gini coefficient** | | **GE (0)** | | **GE (1)** | | **GE (2)** | |
|  | **(1)** | **(2)** | **(3)** | **(4)** | **(5)** | **(6)** | **(7)** | **(8)** |
| Inequality | 0.64** | 0.62*** | 0.36** | 0.32*** | 0.22* | 0.20*** | 0.029 | 0.028 |
|  | (2.17) | (3.77) | (2.51) | (3.61) | (1.80) | (3.01) | (1.09) | (1.46) |
| Region Mean Income | -0.053*** | -0.032*** | -0.050*** | -0.028** | -0.055*** | -0.035*** | -0.059*** | -0.038*** |
|  | (3.79) | (3.14) | (3.21) | (2.51) | (3.59) | (3.17) | (3.17) | (3.14) |
| Household income | |  |  |  |  |  |  |  |
| Q1 |  | -0.039** |  | -0.039** |  | -0.040** |  | -0.0404** |
|  |  | (2.57) |  | (2.51) |  | (2.57) |  | (2.58) |
| Q2 |  | -0.092*** |  | -0.093*** |  | -0.093*** |  | -0.096*** |
|  |  | (7.27) |  | (7.25) |  | (7.37) |  | (7.39) |
| Q3 |  | -0.008 |  | -0.008 |  | -0.008 |  | -0.008 |
|  |  | (0.96) |  | (0.95) |  | (0.95) |  | (0.93) |
| Q4 |  | -0.037*** |  | -0.037*** |  | -0.037*** |  | -0.037*** |
|  |  | (7.95) |  | (7.90) |  | (7.90) |  | (7.88) |
| Q5 |  | -0.004*** |  | -0.003*** |  | -0.003*** |  | -0.003*** |
|  |  | (5.19) |  | (5.18) |  | (5.15) |  | (5.03) |
| Plus department socio-economic development | No | Yes | No | Yes | No | Yes | No | Yes |
| Wald chi-squared | 55 | 22,069 | 59 | 23,208 | 52 | 22,124 | 48 | 27,078 |
| P-value | 0.000 | 0.000 | 0.000 | 0.000 | 0.000 | 0.000 | 0.000 | 0.000 |
| Pseudo R2 | 0.012 | 0.125 | 0.012 | 0.125 | 0.011 | 0.125 | 0.010 | 0.124 |

Note: Absolute t-statistics are reported in parentheses. *** denotes significance at 1%, ** at 5%, and * at 10%. All ordered probit models were estimated with standard errors adjusted for clustering. All estimations include year dummies. Estimations (2), (4), (6) and (8) include individual characteristics and a categorical variable that indicates the level of socio-economic development of the department of residence.

**Table 3.2. Effect of Income Inequality on Individual Health Status: Average Marginal Effects from Probit Models (Urban population)**

|  | **Dependent Variable: 1 if "Fair" or "Poor" reported health status** | | | | | | | | |
| --- | --- | --- | --- | --- | --- | --- | --- | --- | --- |
| **Inequality measure:** | **Gini coefficient** | | **GE (0)** | | | **GE (1)** | | **GE (2)** | |
|  | **(1)** | **(2)** | **(3)** | | **(4)** | **(5)** | **(6)** | **(7)** | **(8)** |
| Inequality |  |  |  | |  |  |  |  |  |
| Q1 | 0.78*** | 0.65*** | 0.52*** | | 0.36*** | 0.38*** | 0.22*** | 0.11*** | 0.029 |
|  | (5.02) | (3.75) | (6.36) | | (3.82) | (5.91) | (2.91) | (4.55) | (1.24) |
| Q2 | 0.62*** | 0.61*** | 0.36*** | | 0.31*** | 0.22*** | 0.18*** | 0.035* | 0.018 |
|  | (3.94) | (3.63) | (4.33) | | (3.48) | (3.42) | (2.63) | (1.86) | (0.96) |
| Q3 | 0.56*** | 0.62*** | 0.30*** | | 0.31*** | 0.17** | 0.20*** | 0.010 | 0.024 |
|  | (3.60) | (3.77) | (3.63) | | (3.49) | (2.56) | (2.98) | (0.59) | (1.43) |
| Q4 | 0.50*** | 0.64*** | 0.24*** | | 0.33*** | 0.10 | 0.23*** | -0.018 | 0.043** |
|  | (3.14) | (3.95) | (2.77) | | (3.69) | (1.54) | (3.45) | (0.97) | (2.25) |
| Q5 | 0.38** | 0.60*** | 0.12 | | 0.28*** | -0.009 | 0.19*** | -0.068*** | 0.027 |
|  | (2.43) | (3.68) | (1.44) | | (3.15) | (0.14) | (2.82) | (3.76) | (1.51) |
| Region Mean Income | -0.034*** | -0.032*** | -0.033*** | | -0.028** | -0.037*** | -0.034*** | -0.043*** | -0.038*** |
|  | (4.19) | (3.12) | (3.78) | | (2.51) | (4.31) | (3.15) | (4.50) | (3.13) |
| Household income |  |  |  | |  |  |  |  |  |
| Q1 |  | -0.039** |  | | -0.038** |  | -0.039** |  | -0.040** |
|  |  | (2.51) |  | | (2.43) |  | (2.52) |  | (2.57) |
| Q2 |  | -0.040* |  | | -0.033 |  | -0.043* |  | -0.066*** |
|  |  | (1.68) |  | | (1.47) |  | (1.89) |  | (3.06) |
| Q3 |  | -0.031 |  | | -0.020 |  | -0.032 |  | -0.028* |
|  |  | (1.49) |  | | (0.94) |  | (1.56) |  | (1.66) |
| Q4 |  | -0.038*** |  | | -0.032*** |  | -0.041*** |  | -0.045*** |
|  |  | (3.91) |  | | (3.45) |  | (4.45) |  | (6.05) |
| Q5 |  | -0.002*** |  | | -0.002*** |  | -0.023*** |  | -0.002*** |
|  |  | (3.23) |  | | (3.10) |  | (3.29) |  | (3.57) |
| Wald chi-squared | 1066 | 31,794 | | 729 | 34,624 | 697 | 32,216 | 369 | 37,918 |
| P value | 0.000 | 0.000 | | 0.000 | 0.000 | 0.000 | 0.000 | 0.000 | 0.000 |
| Pseudo R2 | 0.033 | 0.126 | | 0.032 | 0.126 | 0.031 | 0.125 | 0.028 | 0.124 |

Note: Absolute t-statistics are reported in parentheses. *** denotes significance at 1%, ** at 5%, and * at 10%. All ordered probit models were estimated with standard errors adjusted for clustering. All estimations include year dummies. Estimations (2), (4), (6) and (8) include individual characteristics and a categorical variable that indicates the level of socio-economic development of the department of residence.

**Table 4.1. Effect of Income Inequality on Individual Health Status: Average Marginal Effects from Probit Models (Household heads)**

|  | **Dependent Variable: 1 if "Fair" or "Poor" reported health status** | | | | | | | |
| --- | --- | --- | --- | --- | --- | --- | --- | --- |
| **Inequality measure:** | **Gini coefficient** | | **GE (0)** | | **GE (1)** | | **GE (2)** | |
|  | **(1)** | **(2)** | **(3)** | **(4)** | **(5)** | **(6)** | **(7)** | **(8)** |
| Inequality | 0.40 | 0.55*** | 0.13 | 0.30*** | 0.15 | 0.18** | 0.020 | 0.023 |
|  | (0.79) | (2.99) | (0.54) | (3.03) | (0.76) | (2.42) | (0.55) | (1.19) |
| Region Mean Income | -0.038* | -0.053*** | -0.039* | -0.050*** | -0.039* | -0.056*** | -0.041* | -0.06*** |
|  | (1.87) | (3.91) | (1.87) | (3.47) | (1.82) | (3.92) | (1.68) | (3.81) |
| Household income | |  |  |  |  |  |  |  |
| Q1 |  | -0.084*** |  | -0.083*** |  | -0.085*** |  | -0.087*** |
|  |  | (5.29) |  | (5.28) |  | (5.32) |  | (5.46) |
| Q2 |  | -0.099*** |  | -0.099*** |  | -0.10*** |  | -0.10*** |
|  |  | (6.62) |  | (6.58) |  | (6.67) |  | (6.67) |
| Q3 |  | -0.014 |  | -0.014 |  | -0.014 |  | -0.014 |
|  |  | (1.60) |  | (1.58) |  | (1.60) |  | (1.61) |
| Q4 |  | -0.036*** |  | -0.037*** |  | -0.036*** |  | -0.036*** |
|  |  | (6.07) |  | (6.00) |  | (6.02) |  | (5.94) |
| Q5 |  | -0.003*** |  | -0.003*** |  | -0.003*** |  | -0.003*** |
|  |  | (3.95) |  | (3.94) |  | (3.92) |  | (3.82) |
| Plus department socio-economic development | No | Yes | No | Yes | No | Yes | No | Yes |
| Wald chi-squared | . | 17,629 | . | 17,487 | . | 17,897 | . | 19,582 |
| P-value | . | 0.000 | . | 0.000 | . | 0.000 | . | 0.000 |
| Pseudo R2 | 0.007 | 0.120 | 0.007 | 0.120 | 0.007 | 0.120 | 0.007 | 0.119 |

Note: Absolute t-statistics are reported in parentheses. *** denotes significance at 1%, ** at 5%, and * at 10%. All ordered probit models were estimated with standard errors adjusted for clustering. All estimations include year dummies. Estimations (2), (4), (6) and (8) include individual characteristics and a categorical variable that indicates the level of socio-economic development of the department of residence.

**Table 4.2. Effect of Income Inequality on Individual Health Status: Average Marginal Effects from Probit Models (Household heads)**

|  | **Dependent Variable: 1 if "Fair" or "Poor" reported health status** | | | | | | | |
| --- | --- | --- | --- | --- | --- | --- | --- | --- |
| **Inequality measure** | **Gini coefficient** | | **GE (0)** | | **GE (1)** | | **GE (2)** | |
|  | **(1)** | **(2)** | **(3)** | **(4)** | **(5)** | **(6)** | **(7)** | **(8)** |
| Inequality |  |  |  |  |  |  |  |  |
| Q1 | 0.43* | 0.55*** | 0.22 | 0.31*** | 0.25** | 0.17** | 0.074*** | 0.016 |
|  | (1.66) | (2.90) | (1.56) | (3.00) | (2.39) | (2.07) | (3.05) | (0.68) |
| Q2 | 0.28 | 0.53*** | 0.069 | 0.30*** | 0.093 | 0.16** | 0.003 | 0.014 |
|  | (1.07) | (2.89) | (0.49) | (2.95) | (0.91) | (2.11) | (0.11) | (0.74) |
| Q3 | 0.23 | 0.56*** | 0.025 | 0.300*** | 0.051 | 0.19** | -0.018 | 0.025 |
|  | (0.90) | (3.03) | (0.18) | (2.956) | (0.50) | (2.55) | (0.76) | (1.42) |
| Q4 | 0.19 | 0.61*** | -0.018 | 0.33*** | 0.009 | 0.25*** | -0.037 | 0.057*** |
|  | (0.74) | (3.35) | (0.13) | (3.24) | (0.09) | (3.40) | (1.60) | (3.18) |
| Q5 | 0.078 | 0.55*** | -0.13 | 0.26** | -0.10 | 0.19*** | -0.092*** | 0.037** |
|  | (0.30) | (3.01) | (0.93) | (2.59) | (0.98) | (2.65) | (3.60) | (2.15) |
| Region Mean Income | -0.018 | -0.053*** | -0.020* | -0.050*** | -0.020 | -0.056*** | -0.023* | -0.060*** |
|  | (1.57) | (3.89) | (1.71) | (3.47) | (1.59) | (3.90) | (1.77) | (3.78) |
| Household income |  |  |  |  |  |  |  |  |
| Q1 |  | -0.084*** |  | -0.084*** |  | -0.085*** |  | -0.087*** |
|  |  | (5.29) |  | (5.24) |  | (5.32) |  | (5.49) |
| Q2 |  | -0.08*** |  | -0.078*** |  | -0.086*** |  | -0.096*** |
|  |  | (3.24) |  | (3.31) |  | (3.48) |  | (3.82) |
| Q3 |  | -0.044* |  | -0.027 |  | -0.048** |  | -0.041** |
|  |  | (1.87) |  | (1.14) |  | (2.09) |  | (2.47) |
| Q4 |  | -0.045*** |  | -0.035*** |  | -0.051*** |  | -0.055*** |
|  |  | (3.84) |  | (2.87) |  | (4.34) |  | (6.20) |
| Q5 |  | -0.002** |  | -0.002** |  | -0.002*** |  | -0.002*** |
|  |  | (2.18) |  | (2.11) |  | (2.35) |  | (2.57) |
| Wald chi-squared | 437 | 20,889 | 406 | 22,745 | 395 | 23,090 | 300 | 27,126 |
| P value | 0.000 | 0.000 | 0.000 | 0.000 | 0.000 | 0.000 | 0.000 | 0.000 |
| Pseudo R2 | 0.022 | 0.121 | 0.021 | 0.121 | 0.021 | 0.120 | 0.020 | 0.120 |

Note: Absolute t-statistics are reported in parentheses. *** denotes significance at 1%, ** at 5%, and * at 10%. All ordered probit models were estimated with standard errors adjusted for clustering. All estimations include year dummies. Estimations (2), (4), (6) and (8) include individual characteristics and a categorical variable that indicates the level of socio-economic development of the department of residence.

**Table 5.1. Effect of Income Inequality on Individual Health Status: Average Marginal Effects from Probit Models (Household income without imputed rent and adjusted following SEDLAC)**

|  | **Dependent Variable: 1 if "Fair" or "Poor" reported health status** | | | | | | | | | | |  |
| --- | --- | --- | --- | --- | --- | --- | --- | --- | --- | --- | --- | --- |
| **Inequality measure:** | **Gini coefficient** | | **GE (0)** | | **GE (1)** | | | **GE (2)** | | | |  |
|  | **(1)** | **(2)** | **(3)** | **(4)** | **(5)** | **(6)** | | **(7)** | | **(8)** | |  |
| Inequality | 0.35 | 0.52*** | 0.060 | 0.16** | 0.032 | 0.073* | | -0.004 | | 0.003 | |  |
|  | (0.81) | (2.98) | (0.32) | (2.01) | (0.41) | (1.75) | | (0.97) | | (1.31) | |  |
| Region Mean Income | -0.040* | -0.053*** | -0.0422* | -0.057*** | -0.043* | -0.061*** | | -0.046* | | -0.063*** | |  |
|  | (1.81) | (3.94) | (1.74) | (3.61) | (1.66) | (3.99) | | (1.66) | | (3.94) | |  |
| Household income adj. |  |  |  |  |  | |  | |  | |  | |
| Q1 |  | -0.043*** |  | -0.042*** |  | -0.043*** | |  | | -0.042*** | |  |
|  |  | (2.88) |  | (2.81) |  | (2.86) | |  | | (2.86) | |  |
| Q2 |  | -0.096*** |  | -0.099*** |  | -0.10*** | |  | | -0.10*** | |  |
|  |  | (8.07) |  | (8.03) |  | (7.99) | |  | | (7.99) | |  |
| Q3 |  | 0.005 |  | -0.005*** |  | 0.005 | |  | | 0.005 | |  |
|  |  | (0.75) |  | (0.74) |  | (0.76) | |  | | (0.74) | |  |
| Q4 |  | -0.035*** |  | -0.035*** |  | -0.036*** | |  | | -0.035*** | |  |
|  |  | (7.17) |  | (7.03) |  | (7.15) | |  | | (7.14) | |  |
| Q5 |  | -0.005*** |  | -0.005*** |  | -0.005*** | |  | | -0.05*** | |  |
|  |  | (6.62) |  | (6.60) |  | (6.61) | |  | | (6.58) | |  |
| Plus department socio-economic development | No | Yes | No | Yes | No | Yes | | No | | Yes | |  |
| Wald chi-squared | . | 38,051 | . | 42,669 | . | 40,965 | | . | | 40,864 | |  |
| P-value | . | 0.000 | . | 0.000 | . | 0.000 | | . | | 0.000 | |  |
| Pseudo R2 | 0.008 | 0.121 | 0.007 | 0.121 | 0.007 | 0.120 | | 0.008 | | 0.120 | |  |

Note: Absolute t-statistics are reported in parentheses. *** denotes significance at 1%, ** at 5%, and * at 10%. All ordered probit models were estimated with standard errors adjusted for clustering. All estimations include year dummies. Estimations (2), (4), (6) and (8) include individual characteristics and a categorical variable that indicates the level of socio-economic development of the department of residence.

**Table 5.2. Effect of Income Inequality on Individual Health Status: Average Marginal Effects from Probit Models (Household income without imputed rent and adjusted following SEDLAC)**

|  | **Dependent Variable: 1 if "Fair" or "Poor" reported health status** | | | | | | | |
| --- | --- | --- | --- | --- | --- | --- | --- | --- |
| **Individual income:** | **Gini coefficient** | | **GE (0)** | | **GE (1)** | | **GE (2)** | |
|  | **(1)** | **(2)** | **(3)** | **(4)** | **(5)** | **(6)** | **(7)** | **(8)** |
| Inequality |  |  |  |  |  |  |  |  |
| Q1 | 0.28*** | 0.54*** | 0.22*** | 0.19** | 0.20*** | 0.086* | 0.019*** | 0.005 |
|  | (7.27) | (3.00) | (6.89) | (2.27) | (5.43) | (1.95) | (2.82) | (1.59) |
| Q2 | 0.13*** | 0.51*** | 0.082** | 0.16* | 0.060* | 0.057 | -0.003 | 0.001 |
|  | (3.16) | (2.87) | (2.50) | (1.95) | (1.73) | (1.37) | (0.94) | (0.54) |
| Q3 | 0.082** | 0.51*** | 0.040 | 0.15* | 0.019 | 0.067* | -0.009* | 0.005** |
|  | (1.98) | (2.96) | (1.16) | (1.90) | (0.56) | (1.87) | (1.78) | (2.21) |
| Q4 | 0.018 | 0.54*** | -0.019 | 0.15** | -0.041 | 0.086* | -0.021*** | 0.005 |
|  | (0.44) | (3.12) | (0.55) | (1.97) | (1.15) | (1.90) | (2.80) | (1.32) |
| Q5 | -0.089* | 0.50*** | -0.12*** | 0.12 | -0.14*** | 0.056 | -0.043*** | -0.0002 |
|  | (1.93) | (2.89) | (3.00) | (1.46) | (3.66) | (1.10) | (2.92) | (0.07) |
| Region Mean Income | -0.020 | -0.053*** | -0.020 | -0.057*** | -0.021 | -0.061*** | -0.037*** | -0.064*** |
|  | (1.62) | (3.93) | (1.55) | (3.64) | (1.63) | (3.99) | (2.67) | (3.94) |
| Household income adj. |  |  |  |  |  |  |  |  |
| Q1 |  | -0.042*** |  | -0.041*** |  | -0.042*** |  | -0.042*** |
|  |  | (2.81) |  | (2.75) |  | (2.78) |  | (2.82) |
| Q2 |  | -0.059*** |  | -0.053** |  | -0.061*** |  | -0.088*** |
|  |  | (3.01) |  | (2.45) |  | (3.42) |  | (6.91) |
| Q3 |  | -0.004 |  | 0.006 |  | -0.013 |  | -0.007 |
|  |  | (0.21) |  | (0.35) |  | (0.89) |  | (0.98) |
| Q4 |  | -0.040*** |  | -0.030*** |  | -0.036*** |  | -0.031*** |
|  |  | (3.96) |  | (3.16) |  | (3.20) |  | (5.37) |
| Q5 |  | -0.004*** |  | -0.004*** |  | -0.004*** |  | -0.005*** |
|  |  | (5.36) |  | (5.09) |  | (5.37) |  | (6.10) |
| Wald chi-squared | 659 | 201,450 | 646 | 203,128 | 571 | 136,629 | 171 | 46,967 |
| P value | 0.000 | 0.000 | 0.000 | 0.000 | 0.000 | 0.000 | 0.000 | 0.000 |
| Pseudo R2 | 0.026 | 0.121 | 0.025 | 0.121 | 0.024 | 0.121 | 0.015 | 0.120 |

Note: Absolute t-statistics are reported in parentheses. *** denotes significance at 1%, ** at 5%, and * at 10%. All ordered probit models were estimated with standard errors adjusted for clustering. All estimations include year dummies. Estimations (2), (4), (6) and (8) include individual characteristics and a categorical variable that indicates the level of socio-economic development of the department of residence.
