# Supplementary material for: Income inequality and self-rated health status in Colombia
Source: Int J Equity Health. 2022 May 16;21:69. doi: 10.1186/s12939-022-01659-8 (PMC9108691; doi:10.1186/s12939-022-01659-8)
Supplement: Supplementary file 2 — Additional file 2: Table 6. Effect of Income Inequality on Individual Health Status: Average Marginal Effects from the Probit Multilevel Regressions. Table 7. Effect of Income Inequality on Chronic Heart Disease Status: Average Marginal Effects from Probit Models. Figure 2. Non-linear strong IIH test using restricted cubid splines. [file 12939_2022_1659_MOESM2_ESM.docx]

**Supplemental material – Additional File 2**

**Table 6. Effect of Income Inequality on Individual Health Status: Average Marginal Effects from the Probit Multilevel Regressions**

|  | **Dependent Variable: 1 if "Fair" or "Poor" reported health status** | | | | |
| --- | --- | --- | --- | --- | --- |
| **Inequality measure:** | **Strong IIH** | **Strong IIH with FE by region** | **Strong IIH with RE by region_year** | **Strong IIH with RE by region** | **Strong IIH with RE by region and year** |
| **(A) Gini** | (1) | (2) | (3) | (4) | (5) |
| Inequality | 0.54*** | -0.47*** | 0.73*** | -0.42*** | -0.007 |
|  | (3.00) | (3.52) | (3.18) | (6.93) | (0.03) |
| Wald chi-squared | 32,482 | 40,674 | 28,647 | 28,976 | 28,613 |
| P value | 0.000 | 0.000 | 0.000 | 0.000 | 0.000 |
| Pseudo R2 | 0.120 | 0.128 | . | . | . |
| **(B) GE (0)** | (1) | (2) | (3) | (4) | (5) |
| Inequality | 0.30*** | -0.18** | 0.39*** | 0.16*** | -0.037 |
|  | (3.08) | (2.28) | (3.27) | (5.70) | (0.27) |
| Wald chi-squared | 33,081 | 42,166 | 28,648 | 28,960 | 28,613 |
| P value | 0.000 | 0.000 | 0.000 | 0.000 | 0.000 |
| Pseudo R2 | 0.120 | 0.128 | . | . | . |
| **(C) GE (1)** | (1) | (2) | (3) | (4) | (5) |
| Inequality | 0.18** | -0.16*** | 0.24** | -0.14*** | -0.001 |
|  | (2.44) | (2.78) | (2.54) | (6.99) | (0.01) |
| Wald chi-squared | 34,012 | 38,762 | 28,642 | 28,974 | 28,613 |
| P value | 0.000 | 0.000 | 0.000 | 0.000 | 0.000 |
| Pseudo R2 | 0.120 | 0.128 | . | . | . |
| **(D) GE (2)** | (1) | (2) | (3) | (4) | (5) |
| Inequality | 0.022 | -0.012 | 0.038 | 0.011*** | 0.005 |
|  | (1.14) | (0.79) | (1.58) | (3.44) | (0.27) |
| Wald chi-squared | 36,375 | 53,361 | 28,637 | 28,943 | 28,613 |
| P value | 0.000 | 0.000 | 0.000 | 0.000 | 0.000 |
| Pseudo R2 | 0.120 | 0.128 | . | . | . |

Notes: As presented in Column 3 of Table 6, once we allow for random intercepts by region and year, results are similar, and the coefficients are larger than the main estimates that we present in Column 2 of Table 4 of the manuscript (reproduced in Column 1 of Table 6). However, once we allow for random intercepts of region only, the sign of the coefficient turns negative. This is because ‘random intercepts of region only’ is mainly reflecting the within region variation, rather than the variation of income inequality and health across regions (Colum 4, Table 6). This is also observed when considering region fixed effects, where only within variation is being used (Column 2, Table 6). The main source of variation that we exploit in this paper are the differences across regions, as we are not seeing significant changes in income inequality over the years (for each region). Absolute t-statistics are reported in parentheses. *** denotes significance at 1%, ** at 5%, and * at 10%. All ordered probit models were estimated with standard errors adjusted for clustering. All estimations include year dummies. Estimations (2), (4), (6) and (8) include individual characteristics and a categorical variable that indicates the level of socio-economic development of the department of residence.

**Table 7. Effect of Income Inequality on Chronic Heart Disease Status: Average Marginal Effects from Probit Models**

|  | **Dependent Variable: 1 if "Fair" or "Poor" reported health status** | | | | | | | |
| --- | --- | --- | --- | --- | --- | --- | --- | --- |
| **Inequality measure:** | **Gini coefficient** | | **GE (0)** | | **GE (1)** | | **GE (2)** | |
|  | **(1)** | **(2)** | **(3)** | **(4)** | **(5)** | **(6)** | **(7)** | **(8)** |
| Inequality | -0.17 | 0.28 | -0.18 | 0.19 | -0.062 | 0.062 | -0.017 | 0.006 |
|  | (0.40) | (0.84) | (0.79) | (1.12) | (0.40) | (0.50) | (0.49) | (0.23) |
| Region Mean Income | 0.023 | -0.020* | 0.022 | -0.018 | 0.024 | -0.021* | 0.023 | -0.022* |
|  | (1.50) | (1.72) | (1.55) | (1.51) | (1.45) | (1.72) | (1.39) | (1.76) |
| Household income | |  |  |  |  |  |  |  |
| Q1 |  | 0.007 |  | 0.008 |  | 0.007 |  | 0.007 |
|  |  | (0.32) |  | (0.37) |  | (0.31) |  | (0.30) |
| Q2 |  | -0.023 |  | -0.023 |  | -0.025 |  | -0.028 |
|  |  | (1.33) |  | (1.26) |  | (1.39) |  | (1.44) |
| Q3 |  | 0.010 |  | 0.010 |  | 0.010 |  | 0.010 |
|  |  | (0.86) |  | (0.86) |  | (0.87) |  | (0.88) |
| Q4 |  | 0.004 |  | 0.004 |  | 0.004 |  | 0.004 |
|  |  | (1.04) |  | (1.06) |  | (1.05) |  | (1.04) |
| Q5 |  | -0.001** |  | -0.001** |  | -0.001** |  | -0.001** |
|  |  | (2.45) |  | (2.49) |  | (2.35) |  | (2.21) |
| Plus department socio-economic development | No | Yes | No | Yes | No | Yes | No | Yes |
| Wald chi-squared | 616 | 174,724 | 496 | 351,592 | 753 | 387,498 | 981 | 1.23×10^7^ |
| P-value | 0.000 | 0.000 | 0.000 | 0.000 | 0.000 | 0.000 | 0.000 | 0.000 |
| Pseudo R2 | 0.007 | 0.116 | 0.007 | 0.117 | 0.007 | 0.116 | 0.007 | 0.124 |

Note: Absolute t-statistics are reported in parentheses. *** denotes significance at 1%, ** at 5%, and * at 10%. All ordered probit models were estimated with standard errors adjusted for clustering. All estimations include year dummies. Estimations (2), (4), (6) and (8) include individual characteristics and a categorical variable that indicates the level of socio-economic development of the department of residence.

| **Figure 2**. Non-linear strong IIH test using restricted cubid splines  Dependent Variable: 1 if "Fair" or "Poor" reported health status | |
| --- | --- |
| 1. Gini coefficient | |
| $\Pr\left( H=1 \vert Gini,X \right)$ vs $Gini$  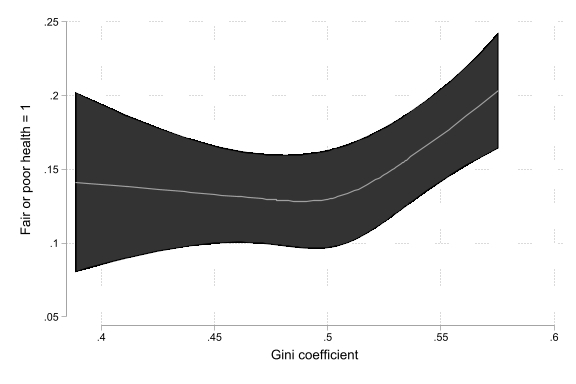 | $\partial\Pr\left( H=1 \vert Gini,X \right)/\partial Gini$ vs $Gini$  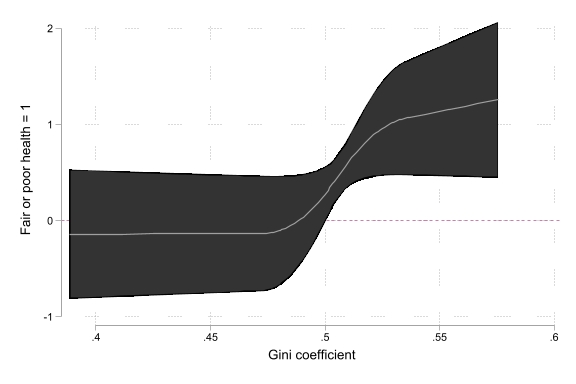 |
| 1. GE(0) | |
| $\Pr\left( H=1 \vert GE\left( 0 \right),X \right)$ vs $GE\left( 0 \right)$  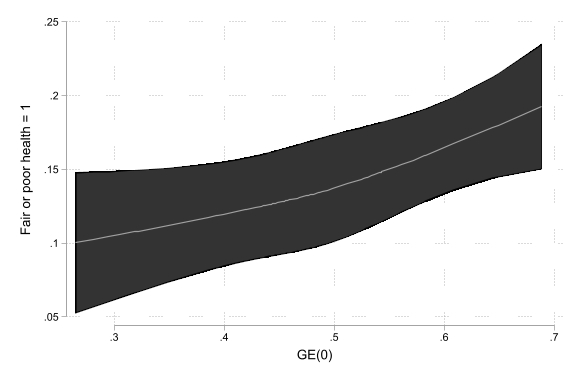 | $\partial\Pr\left( H=1 \vert GE\left( 0 \right),X \right)/\partial GE\left( 0 \right)$ vs $GE\left( 0 \right)$  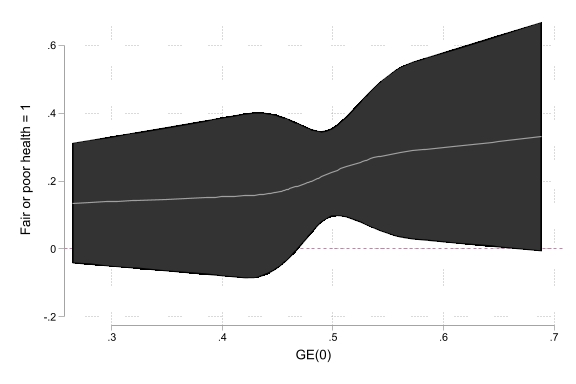 |
| 1. GE(1) | |
| $\Pr\left( H=1 \vert GE\left( 1 \right),X \right)$ vs $GE\left( 1 \right)$  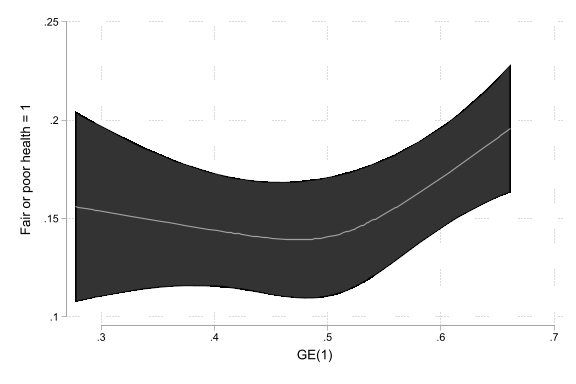 | $\partial\Pr\left( H=1 \vert GE\left( 1 \right),X \right)/\partial GE\left( 1 \right)$ vs $GE\left( 1 \right)$  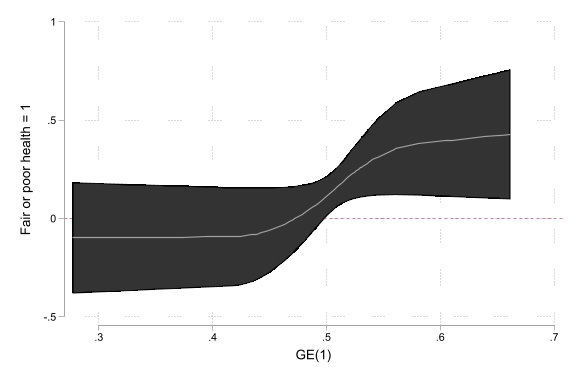 |
| 1. GE(2) | |
| $\Pr\left( H=1 \vert GE\left( 2 \right),X \right)$ vs $GE\left( 2 \right)$  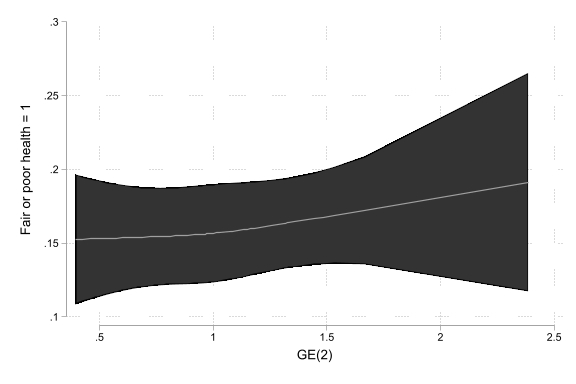 | $\partial\Pr\left( H=1 \vert GE\left( 2 \right),X \right)/\partial GE\left( 2 \right)$ vs $GE\left( 2 \right)$  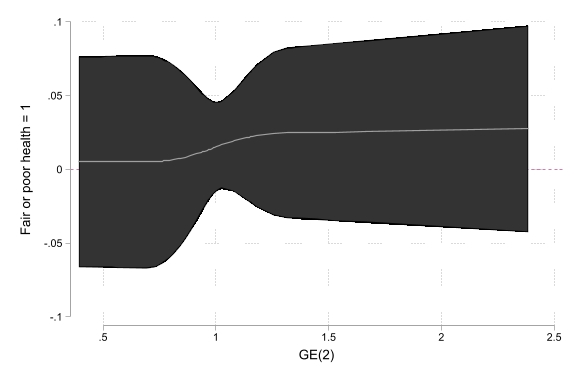 |
| Notes: Graphs present predicted values of the dependent variable for different values of the inequality measures (left), and the corresponding marginal effect. All of them were calculated after estimating probit regressions where the inequality measure was included as a cubic spline with three knots. | |
